# Breaking Down SERS Detection Limit: Engineering of a Nanoporous Platform for High Sensing and Technology

**DOI:** 10.3390/nano12101737

**Published:** 2022-05-19

**Authors:** Federico Scaglione, Livio Battezzati, Paola Rizzi

**Affiliations:** Dipartimento di Chimica and Centro Interdipartimentale NIS (Nanostructured Interfaces and Surfaces), Università di Torino, V. Giuria 7, 10125 Turin, Italy; livio.battezzati@unito.it (L.B.); paola.rizzi@unito.it (P.R.)

**Keywords:** nanoporous gold, anodization, chemical dealloying, amorphous precursor, SERS, electrocatalyst

## Abstract

In this study, nanoporous gold (NPG) was synthesized by free corrosion dealloying of an amorphous precursor, Au_20_Cu_48_Ag_7_Pd_5_Si_20_ (at. %), in a mixture of nitric and hydrofluoric acid, starting from amorphous melt-spun ribbons. NPG revealed a 3D nanoporous structure composed of pores and multigrain ligaments of an average size of 60 nm. NPG was further anodized in oxalic acid at 8 V vs. Ag/AgCl reference electrode to obtain a bimodal morphology composed of ligaments disrupted in finer features. Both NPG and anodized samples (A-NPG) were found to be mechanically stable to bending and active for surface-enhanced Raman scattering (SERS). SERS activity of samples was investigated using 4,4′-bipyridine as a probe molecule. A detection limit of 10^−16^ M was found for both samples, but in A-NPG, the signal was strongly enhanced. The extremely high enhancement obtained for A-NPG is attributed both to the small size of ligaments and crystals of which they are made, as well as to the nanometric features resulting from anodization treatment. Such a microstructure showed homogenous SERS response in terms of average enhancement all across the surface, as demonstrated by mapping measurements. Furthermore, NPG and A-NPG were tested as electrodes for electrocatalytic applications, showing good properties. The engineering steps from the amorphous precursor to A-NPG led us to obtain a high-sensing platform, with extremely low detection limit and intrinsic properties, that might significantly contribute to the cutting-edge technology of the future.

## 1. Introduction

Studies on nanostructured materials are progressively increasing year by year, due to their unique properties, completely different from those of their bulk counterparts.

In particular, nanoporous metals prepared by dealloying techniques have been receiving research interest and exhibit promising results in several fields of application: actuators [1], storage and conversion of energy [2], electrodes for electrocatalysis [3,4], electrochemical biosensors [5,6,7], and surface-enhanced Raman scattering [8,9]. Scattering enhancement has been ascribed to their high surface area and their morphology constituted in ligaments, ranging from tens to hundreds of nanometers, and basically composed of the noblest elements of the original alloy. Dimensions of pores and ligaments can be tuned by selecting a proper alloy composition and changing dealloying parameters [10]. Furthermore, previous studies demonstrated how the original structure of the precursor, i.e., crystalline or amorphous, affects the morphology and microstructure of ligaments and related properties. When a crystalline alloy is dealloyed, the nanoporous material that is formed retains the crystal structure of the precursor: Lesser noble elements are dissolved into the electrolyte, while the nobler element diffuses by surface diffusion forming ligaments with a smooth appearance [11]. In the case of an amorphous precursor, ligaments are made up of many nanocrystals [12] originated by the germination during the transition from amorphous to crystalline structure [13,14]: When lesser noble atoms of the amorphous alloy are dissolved in the electrolyte, more noble atoms are freed by lateral coordination and move as ad-atoms by surface diffusion forming clusters that grow in dimension with the proceeding of the process until they impinge together forming ligaments. As a result, ligaments obtained from the dealloying of an amorphous precursor are multigrained, with more active sites at the grain boundaries and defects, making them more favorable in electrocatalysis applications [15,16,17] and surface-enhanced Raman spectroscopy (SERS) [18].

SERS is a technique based on the enhancement of light Raman scattering of molecules adsorbed on plasmonic metallic nanostructures [19,20]; nanostructures can be self-standing or supported on oxides as in the case of metallic nanoparticles [21]. The SERS activity of different substrates can be compared according to the enhancement factor (EF), which, consequently, may affect the limit of detection (LOD), i.e., the lowest amount or concentration detectable on the substrate for a given analyte [22]. EF changes by different orders of magnitude depending on the interaction between substrate and molecules (chemical enhancement) and on the nanostructured surface (electromagnetic field enhancement): The former can result in an EF change of two orders of magnitude [23,24], while the latter arises from the resonant excitation of localized surface plasmons on the metallic surface and dramatically raises the EF [25]. The electromagnetic field enhancement is strongly connected to specific features and dimensions of the nanostructures, i.e., sharp edges and tips [26], inter-particle gaps [27], and nanopores [28], typically defined as “hot spots”. Due to this behavior, SERS represents a sensitive technique to reveal molecules of analyte in low quantities that can be improved through the optimization of metallic nanostructured substrates.

In the frame of this application, in this study, a sensor–substrate platform for SERS was investigated, which was obtained by dealloying an Au_20_Cu_48_Ag_7_Pd_5_Si_20_ amorphous ribbon. The resulting nanoporous gold (NPG) was then electrochemically treated via anodization, forming tiny features on NPG ligaments: The final sample was constituted by anodized nanoporous gold (A-NPG) and exhibited a higher SERS activity, as it was able to reveal probe molecules in extremely low concentration, i.e., 10^−16^ M with 4-4′ bipyridine. Once the extremely high SERS activity of the A-NPG sample was proven with a probe molecule, measurements were provided with a molecule of interest whose affinity with the substrate was considered weaker than previous probe molecules: Ascorbic acid was selected due to the need for measuring this molecule in a very low concentration since it is involved in several biological processes or widely used in the pharmaceutic field and in the food industry as a nutritional supplement and food preservative. Furthermore, since the samples were both NPG and A-NPGs self-standing materials, these samples were successfully tested as electrodes for electrocatalytic applications using the same molecule.

## 2. Materials and Methods

Lumps of pure elements (Au: 99.99%, Si: 99.9995%, Ag, Cu, Pd: 99.99%) were arc-melted in a Ti-gettered Ar atmosphere to obtain a master alloy ingot of composition Au_20_Cu_48_Ag_7_Pd_5_Si_20_ (at. %). The ingot was then placed in a quartz crucible, melted via induction, and rapidly quenched via the melt-spinning technique; the rapid solidification process consists of spinning the molten alloy onto a copper wheel, rotating at a speed rate of 25 m/s. The heat of the molten alloy is rapidly dissipated by the wheel, and solidification occurs in form of ribbons, skipping crystallization. Ribbons were 2 mm wide, 20–25 μm thick, and appeared to be fully amorphous according to the X-ray diffraction (XRD) analysis. Dealloying of ribbons was performed by chemical means in a mixture of 10 M HNO_3_ + 0.5 M HF at 70 °C for 4 h; HF was added to avoid the formation of SiO_2_ when the contained silicon in the alloy precursor is oxidized. These conditions were optimized in our previous research to obtain the desired NPG morphology [29]. Pieces of NPG ribbon, 3 cm in length, were subsequently anodized in 0.3 M oxalic acid solution while applying a potential of 8 V for 3, 5, and 7 min. Samples were used as working electrodes in a cell composed of an Ag/AgCl reference electrode in a double-bridge configuration and a Pt-grid counter electrode.

As prepared ribbons, NPG and anodized samples (A-NPG) were properly characterized. The microstructure and structure of dealloyed samples were studied via scanning electron microscopy (SEM) with energy-dispersive X-ray spectroscopy (EDS) after Co calibration and X-ray diffraction (XRD) in Bragg–Brentano geometry with Cu-Kα radiation. Ligaments’ size was measured at their narrower necks using Leica software [30]. All samples were deeply rinsed with ultrapure water to remove the excess acid solution inside pores and then air-dried before electrochemical or SERS experiments.

Electrocatalytic properties toward oxidation of ascorbic acid (AA) were studied using the same experimental setup as that applied for anodization. A buffer solution of 0.1 M KH_2_PO_4_ with 0.02 M ascorbic acid was used as the electrolyte. Current densities have been normalized by making use of the electrochemically active surface area of the electrode [31,32]. Micro-Raman measurements were performed with a Renishaw inVia Raman Microscope using a 785 nm laser line with an acquisition time of 20 s, an accumulation of 10 spectra, 0.05% power at the sample, and a 50× objective; 4,4′-bipyridine was chosen as probe molecules for SERS experiments.

NPG and A-NPG samples were immersed in an ethanol solution of 4,4′-bipyridine with a concentration from 10^−16^ M to 10^−12^ M overnight, enabling the probe molecules to be adsorbed on the surface. Measurements were performed on the sample after drying in air, acquiring random spots on the surface or maps in contiguous areas. SERS intensity mapping image of a 20 × 24 μm^2^ area with a step length of 2 μm was collected using a 4,4′-bipyridine concentration of 10^−12^ M through monitoring the characteristic peak of the probe molecule at 1619 cm^−1^. All solutions were prepared from chemical-grade reagents and deionized water. The spectrometer was calibrated before measurements using the Raman band of a silicon wafer at 520 cm^−1^.

## 3. Results and Discussion

**Figure 1:**
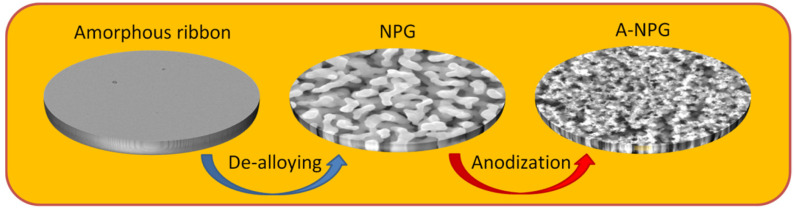
Engineering step procedure for A-NPG: the amorphous ribbon is dealloyed to obtain NPG. Then, anodization of NPG in 0.3 M oxalic acid solution leads to anodized NPG.

The engineering steps to synthesize the A-NPG platform are reported as follows (the step procedure is schematized in Figure 1): NPG was obtained by free dealloying of the amorphous precursor in 10 M HNO_3_ + 0.5 M HF at 70 °C for 4 h; then, it was anodized in 0.3 M oxalic acid solution applying a potential of 8 V (vs. Ag/AgCl) for different times. In what follows, the full characterization of NPG and A-NPG samples is reported.

### 3.1. Morphology Evolution during Anodization Treatment

**Figure 2:**
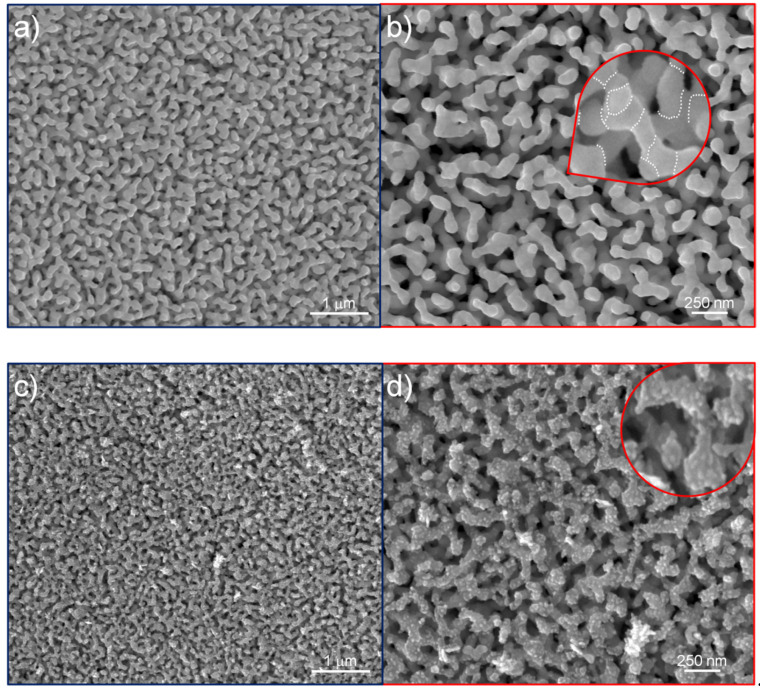

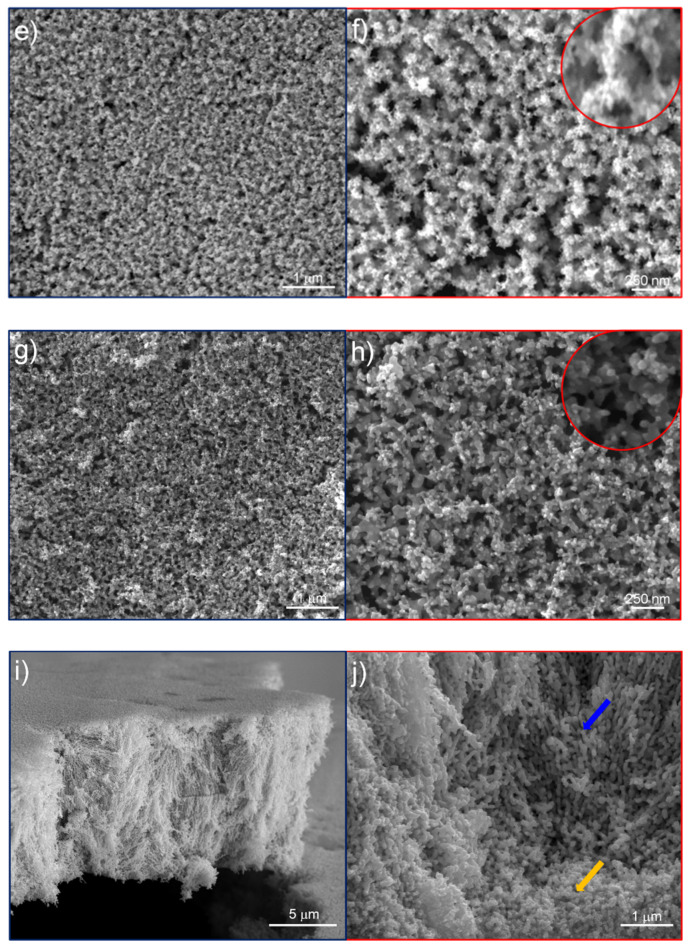
SEM images of the surface of samples: (**a**,**b**) NPG; (**c**,**d**) 3 min A-NPG; (**e**,**f**) 5 min A-NPG; (**g**,**h**) 7 min A-NPG. In these insets, an increased level of detail of ligaments is shown; (**i**) cross-sectional view of NPG; (**j**) cross-sectional view of the 5 min A-NPG. Anodization involved only a few microns on the nanoporous structure inside the thickness of the ribbon (yellow arrow); the inner part of the ribbon was indeed untouched by the treatment (blue arrow).

After dealloying, the nanoporous structure presented as a network of ligaments and pores spread over the whole thickness of the ribbon (Figure 2a,b,i). The average size of ligaments was evaluated as 60 nm, measured on the ligament’s neck. Ligaments are polycrystalline, with grain boundaries joining different crystals, as evidenced by dot lines in the inset of Figure 2b; such a morphology results during the dealloying of an amorphous precursor [14], where the mechanism of dissolution of the lesser noble elements and diffusion of the noble one creates gold nanocrystals impinged together, forming ligaments. After 3 min of anodization, the A-NPG sample showed (Figure 2c,d and inset) an increased roughness constituted by asperities and features (less than 10 nm) randomly formed on the surface during the treatment.

In the sample anodized for 5 min (5 min A-NPG), the roughness evolved in several pointed regions and tips of roughly 10 nm (Figure 2e,f and inset). After increasing the time of anodization to 7 min, the roughness seemed to be reduced in smoothed particles ranging from 20 to 40 nm in size (Figure 2g,h and inset). 

The cross-sectional view for anodized samples (Figure 2j) displayed a morphology in the external part of the ribbon that was affected by the anodization treatment (see yellow arrow), whereas when observing the inside of the section thickness, the NPG morphology was maintained (see blue arrow). EDS analyses performed on NPG samples showed that ligaments were composed of almost pure gold (96.9 at. %), with impurities of copper (0.3 at. %), silver (1.2 at. %), silicon (1.5 at. %), and palladium (0.1 at. %) as remains of the dealloying process. As reported elsewhere [33], retained atoms, silver, in particular, are supposed to contribute to the SERS enhancement.

During anodization, the NPG surface undergoes electro-oxidation [34]; however, this gold oxide is easily reduced to zero-valent gold by oxalic acid, as reported elsewhere [35].

**Figure 3:**
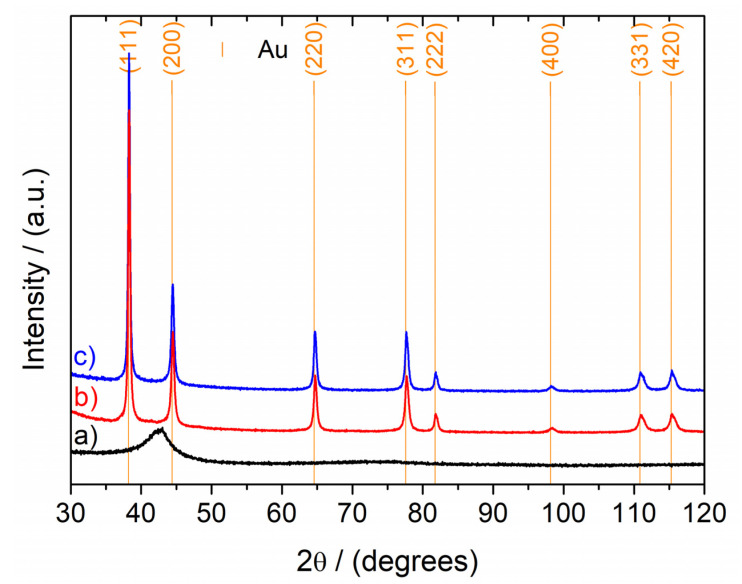
XRD patterns of the as-spun ribbon, NPG, and 3 min A-NPG. The amorphous halo of the as-quenched ribbon (**a**) disappeared after dealloying treatment, while Au fcc reflections appeared on the NPG (**b**) and A-NPG (**c**) patterns.

XRD patterns of the as-spun ribbon, NPG, and 3 min A-NPG are reported in Figure 3. The amorphous halo of the precursor disappeared after the full dealloying of the ribbon, while reflections of the Au fcc increased in the NPG pattern. As expected, the pattern of the 3 min A-NPG sample did not show significant differences from that of the previous one.

### 3.2. Mechanical Stability and Electrocatalytic Properties

NPG and A-NPG present unique hallmarks with respect to the SERS substrates currently on the market (Ocean Optics [36], Hamamatsu [37]). 

Commercial substrates are categorized into two groups: patterned, metallic nanostructures formed by nanoimprint and laser technology, and metallic (Au, Ag) nanoparticles supported by oxides. Both are assembled on a handling plate material of larger dimensions relative to the active area, which limits their versatility of use.

NPG and A-NPG are self-standing, mechanically stable, and flexible if handled with laboratory tweezers; this makes the integration of the substrate into a handling plate unnecessary, widening their range of application in different operative conditions. The flexibility and bendability of samples can be observed in photos shown in Figure S1, Supplementary Materials.

Indeed, the ribbon shape makes them extremely practical for solution measurements, at air after incubation, and in the cuvette. As electrodes, NPG and A-NPG take advantage of the mechanical stability and can be incorporated into sensors and small devices. Another important aspect concerns the possibility to reuse the substrate, something not ever foreseen by other substrates on the market. In fact, NPG can be reused up to twenty times [5] and A-NPG twice, keeping its sensitivity unchanged after washing in piranha solution (70% sulfuric acid, 30% hydrogen peroxide) and abundant rinsing in deionized water until neutrality.

**Figure 4:**
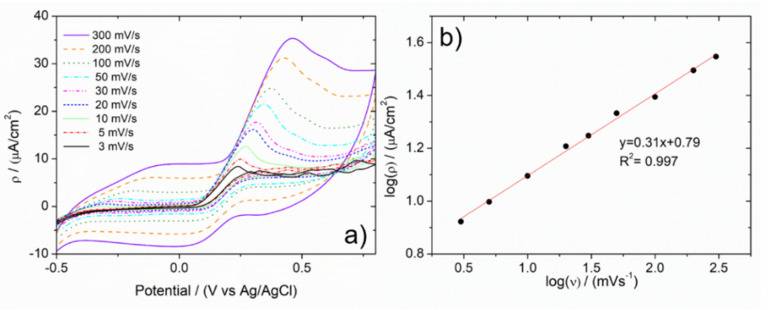
(**a**) CV scans of 0.02 M ascorbic acid in 0.1 M KH_2_PO_4_ solution at 3 min A-NPG electrode as a function of scan speed; (**b**) plot log(ρ) vs. log(ν) showing linearity trend and diffusive control process.

Electrocatalytic properties were studied in the case of the electrochemical oxidation of ascorbic acid (AA) using A-NPG as a working electrode in a cell composed of a Pt counter electrode and a saturated Ag/AgCl reference electrode in a double-bridge configuration. A buffer solution of 0.1 M KH_2_PO_4_ with 0.02 M ascorbic acid was used as the electrolyte. In Figure 4, CV scans at different scan speeds are reported. The oxidative current of the AA increases as a function of the scan speed due to the heterogeneity of the system. Plotting the intensity of the current density vs. the scan speed, both on a logarithmic scale, revealed a linear trend (Figure 4b), suggesting that the process is under diffusive control [38]. Similar results were obtained with NPG, in the same experimental conditions. Furthermore, in that case, a calibration line was obtained by scanning the electrode in the same buffer solution but at different concentrations of AA. Then, the capability of the electrode was verified in the determination of the analyte in a real sample; a Cebion Vitamin C Orange Effervescent tablet of 1000 mg AA (Bracco) was directly dissolved in a proper amount of buffer solution. The oxidative current density registered in such a solution was reported in terms of concentration, followed by grams per tablet of analyte; owing to the former calibration line, recovery concentration and grams content were in good agreement with the package leaflet.

### 3.3. SERS Activity

**Figure 5:**
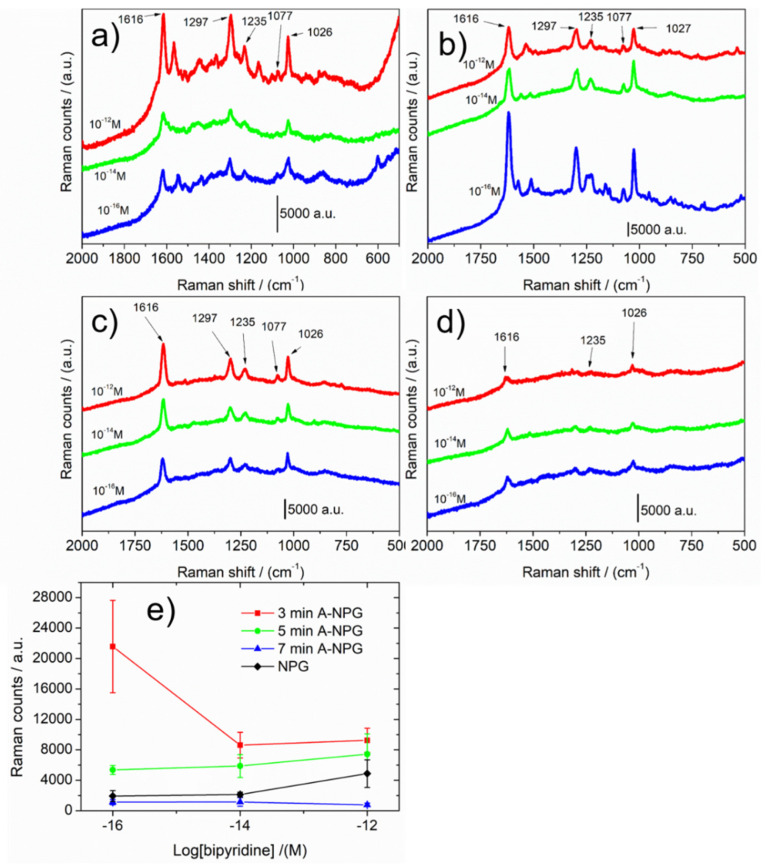
The highest SERS spectra (average of 10 accumulations) of 4,4′-bipyridine at different concentrations on (**a**) NPG, (**b**) 3 min A-NPG, (**c**) 5 min A-NPG, and (**d**) 7 min A-NPG; (**e**) Raman intensity at 1616 cm^−1^ versus bipyridine concentrations (on logarithmic scale). The error bars were calculated from at least five measurements on random spots on the same substrate.

SERS activities of NPG and A-NPG were measured by dipping samples in 4-4′-bipyridine ethanol solution in diluted concentration (i.e., 10^−12^ M, 10^−14^ M, 10^−16^ M). Figure 5a–d shows the highest spectra collected for NPG, 3 min A-NPG, 5 min A-NPG, and 7 min A-NPG, respectively. Spectra show the main signals attributed to the molecule in agreement with the literature [39,40]. The enhancement of the signal was observed in all samples, but it strongly improved, especially at 10^−16^ M, in the 3 min A-NPG sample: the average intensity of the signal as a function of bipyridine concentration was 11 times higher, compared with the one of the NPG sample. This value is extremely interesting, considering that SERS substrates currently on the market (i.e., supported Au/Ag nanoparticles, lithographed Au) report a detection limit in the ppm–ppb range, using probe molecules; therefore, no more than 10^−9^ M and five orders of magnitude smaller than 10^−16^ M was obtained with 3 min A-NPG sample. A stronger signal when the concentration is lower might be assigned to a hot spot, which is randomly distributed and located on the surface. 

In the literature, a similar detection limit is reported with rhodamine 6G as a probe molecule, on complex structures constituted by silver nanoparticles decorated with zinc oxide/silicon heterostructured arrays [41] or three-dimensional sunflower-like nanoarrays decorated with Ag nanoparticles [42]. However, their expensive and time-consuming methods of preparation represent a significant drawback for large-scale applications.

The higher sensibility of A-NPGs is due to the finer double-nanostructured morphology of samples: ligaments of 60 nm with smaller features obtained by the anodization treatment. Researchers believe that the plasmonic effect of nanostructured metallic surfaces is divided into two contributions—the chemical enhancement due to the charge transfer between adsorbed molecules and substrate and electromagnetic enhancement due to the morphology and microstructure of the substrate. Considering that the former contributes two orders of magnitude to the whole enhancement, the latter contributes considerably, demonstrating the higher sensibility of the A-NPG. The enhancement factor (EF) of NPG, 3 min A-NPG, 5 min A-NPG, and 7 min A-NPG was calculated according to the calculation formula of [43] as 2 × 10^13^, 1 × 10^17^, 3 × 10^16^, and 6 × 10^15^, respectively. 

**Figure 6:**
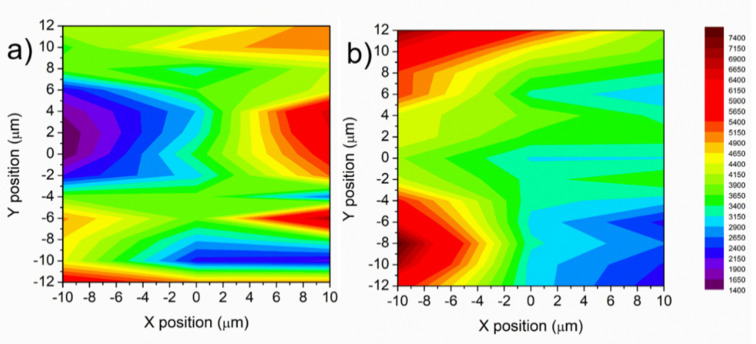
SERS intensity map image of 20 × 24 µm^2^ by lateral step of 2 µm with 4-4′ bipyridine concentration of 10^−12^ M based on characteristic peak at 1616 cm^−1^ on (**a**) NPG and (**b**) 3 min A-NPG. The reader is referred to the web version of this article for interpretation of the references to color in this figure legend.

Figure 6a,b report SERS intensity mapping images with bipyridine concentration of 10^−12^ M on NPG and 3 min A-NPG, respectively. The intensity of the signal was not constant along the surface due to the presence of “hot spots” distributed at random (red regions of the maps). As already observed in a previous study [44], the spot-to-spot intensity variation can be attributed to the localized variation of the electromagnetic field at each “hot spot”, as well as the dynamics and unique environment of a single molecule [26].

However, in the 3 min A-NPG, the average intensity was more shifted in the yellow-red intensity scale bar (Figure 6b), meaning a general sensing improvement of the substrate after anodization.

**Figure 7:**
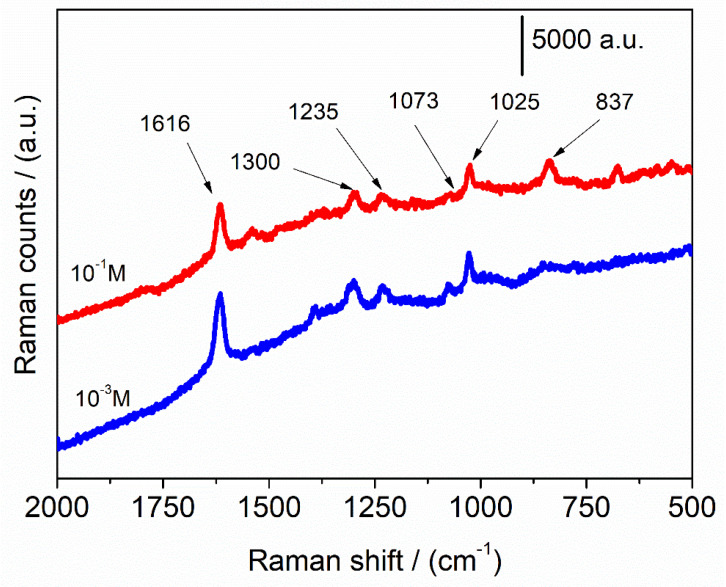
The highest SERS spectra (average of 10 accumulations) of ascorbic acid at different concentrations on 3 min A-NPG. Spectra were acquired randomly on the surface.

Detection of molecules of interest was successfully attempted for NPG with melamine approaching a detection limit of 10^−6^ M [29]. In Figure 7, the SERS activity of 3 min A-NPG tested in ascorbic acid aqueous solution is reported; a LOD of 10^−3^ M was measured for the anodized sample, while NPG did not show any interaction with the analyte and the spectrum, not reported, is a flat line. This further confirmed the higher sensitivity of the A-NPG sample.

## 4. Conclusions

A nanoporous gold obtained by free dealloying of an amorphous precursor was successfully anodized in 0.3 M oxalic acid solution at 8 V (vs. Ag/AgCl) for different times. The ligament morphology evolved from the NPG sample to the A-NPG, increasing the roughness and forming asperities and tips as a function of the time: after three minutes of anodization, roughness and features 5–10 nm in size were formed on ligaments; in contrast, when the treatment was prolonged for 7 min, a smoothening effect prevailed, and roundish smoothed particles of 20–40 nm in size were formed. 

NPG and A-NPG samples result in an easy-to-handle, self-standing material, well-suited as an electrode for electrocatalysis and substrate for SERS.

Electrocatalytic performances were proved by studying the electro-oxidation of ascorbic acid in a buffer solution: CV scan plots showed a signal associated with the oxidative current density of the analyte, increasing as a function of the scan speed and suggesting that the process was under diffusive control. 

SERS activity was attempted first with a probe molecule, 4-4′ bipyridine, reaching a LOD of 10^−16^ M, and for 3 min A-NPG sample, an intensity 11 times higher compared with that of the NPG. Higher activity of 3 min A-NPG was ascribed to the localized enhanced electromagnetic fields at nanosized ligaments and features obtained after anodization. A trial with ascorbic acid in an aqueous solution showed a SERS signal of the molecule down to 10^−3^ M. These results revealed that this anodized nanoporous gold can be successfully applied as an ultrasensitive sensor–substrate platform for SERS and as an electrode for catalysis or chemical and biological analyses.

## 5. Patents

This paper is based on the in-print Italian patent number 102020000024382 filed on 15 October 2020 and patent number PCT/IB2021/059525 filed on 15 October 2021 [45,46].

## Data Availability

The raw/processed data required to reproduce these findings cannot be shared at this time, as the data also form part of an ongoing study.

## Supplementary Materials

The following supporting information can be downloaded at: https://www.mdpi.com/article/10.3390/nano12101737/s1, Figure S1: Photo of 3 min A-NPG bent and kept with tweezers. A-NPG is mechanically stable.
