# Supplementary material for: Breaking Down SERS Detection Limit: Engineering of a Nanoporous Platform for High Sensing and Technology
Source: Nanomaterials (Basel). 2022 May 19;12(10):1737. doi: 10.3390/nano12101737 (PMC9143797; doi:10.3390/nano12101737)
Supplement: Supplementary file 1 [file nanomaterials-12-01737-s001.zip › nanomaterials-1668124-supplementary.pdf]

Supplementary Materials

Breaking down SERS Detection Limit: Engineering of a Nanoporous Platform for High Sensing and Technology

Federico Scaglione *, Livio Battezzati and Paola Rizzi

Dipartimento di Chimica and Centro Interdipartimentale NIS (Nanostructured Interfaces and Surfaces), Università di Torino, V. Giuria 7, 10125 Turin, Italy; livio.battezzati@unito.it (L.B.); paola.rizzi@unito.it (P.R.)

* Correspondence: federico.scaglione@unito.it

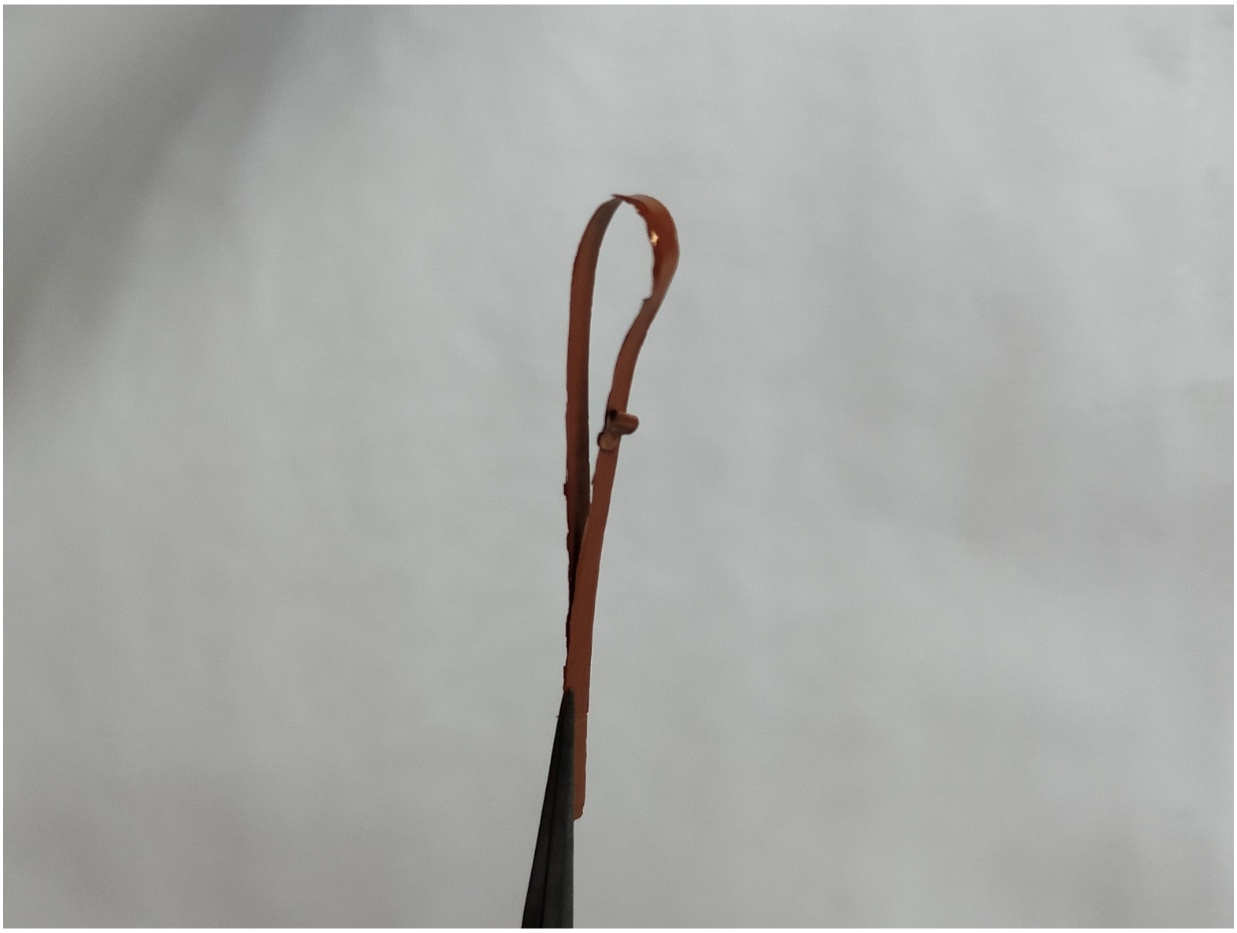

Figure S1. Photo of 3 min A-NPG bent and kept with tweezers. A-NPG is mechanically stable.
